# Competence narrowing in AI-assisted oral English instruction: A quasi-experimental study of the spiral synergy framework

**DOI:** 10.1371/journal.pone.0357515

**Published:** 2026-10-27

**Authors:** Jinyan Zhang

**Affiliations:** School of Economics and Management, Jiangsu Maritime Institute, Nanjing, Jiangsu, China; Universitas Galuh Ciamis, INDONESIA

## Abstract

The rapid integration of generative AI tools into spoken English instruction has produced measurable fluency and accuracy gains, but whether these are evenly distributed across the dimensions of communicative competence remains underexplored. This quasi-experimental study compares the Spiral Synergy Framework (SSF), a three-layer pedagogical sequence (AI Consolidation, Bridging Tasks, Interpersonal Activation), against conventional AI-integrated instruction in two intact Business English classes at a Chinese higher vocational college (experimental n = 36, control n = 34), using oral recordings at three time points scored on five competence dimensions, 15 weeks of classroom observation, and an intra-rater reliability check. The two classes differed significantly at baseline on the oral composite score and three of five dimensions (control higher), though not on CET-4 proficiency or questionnaire items. Because treatment is confounded with class membership and both classes received AI-assisted practice, findings are reported as associations rather than demonstrated causal effects. Linear mixed-effects models testing Group × Time interactions across five outcomes (four dimensions and the composite), corrected for multiple comparisons, identified one outcome — real-time responsiveness — on which the classes diverged: the experimental class maintained responsiveness across the semester while the control class declined despite its baseline advantage (Benjamini–Hochberg-adjusted p = .029), a result that held under a cluster-robust sensitivity check. No corrected between-group difference was found for improvisation, coherence, naturalness, or the composite score; improvisation declined descriptively in both classes over the semester. Classroom observation data (experimental class only) document a recurring, non-monotonic cycle of AI-dependency reduction and rebound across 15 weeks. Intra-rater test–retest reliability of the oral-competence ratings was moderate to good (ICC = .67–.91), though this reflects a single rater familiar with participants rather than independent inter-rater agreement. Implications for multidimensional assessment, task sequencing, and cautious interpretation of AI-integrated curriculum research are discussed.

## Introduction

Generative AI tools for oral English practice—ChatGPT, Speak, ELSA, and similar platforms—have entered EFL classrooms at considerable speed, with early evidence broadly encouraging: improvements in pronunciation accuracy, reductions in speaking anxiety, and increases in output volume are well documented [1–3]. What remains largely untested is whether these gains are evenly distributed across the dimensions of communicative competence, or concentrated in the dimensions AI tools are best equipped to develop—leaving others comparatively under-supported.

A recurring pattern in the author’s own Business English instruction motivates this inquiry: students whose AI-assisted output became measurably more fluent did not always become more communicative. Eye contact faded; repair strategies atrophied; improvisation declined. When AI tools were removed, many students experienced elevated difficulty—yet also came alive in ways that polished AI-assisted performance had not produced.

The present study tests the hypothesis—termed competence narrowing—that sustained AI-mediated oral practice produces gains concentrated in formal and discourse dimensions while strategic competence and improvisational ability show comparatively weaker gains or decline. Rather than asserting this as an established phenomenon, the study treats it as a testable empirical pattern. In response to this hypothesised asymmetry, the Spiral Synergy Framework (SSF) is proposed and tested: a three-layer pedagogical sequence (AI Consolidation → Bridging Tasks → Interpersonal Activation) designed to structure AI-mediated and interpersonal practice as functionally distinct and complementary.

Two research questions organise the inquiry:

RQ1: Does a quasi-experimental comparison of two intact EFL classes reveal evidence of competence narrowing—dissociation between composite oral competence gains and gains in improvisation and real-time responsiveness—over one semester?

RQ2: Does the SSF produce measurable differences in this pattern relative to a control class receiving conventional AI-assisted instruction, and if so, are these differences stable or subject to recurring fluctuation?

## Theoretical framework and literature review

The pedagogical promise of generative AI in oral English instruction rests on an untested assumption: that gains on measurable dimensions of performance translate into gains in communicative competence as a whole. This assumption matters practically as well as theoretically. Vocational and university programmes are rapidly integrating AI speaking tools into curricula on the strength of fluency and pronunciation gains reported in the literature [1–3]; if these gains are concentrated in a subset of competence dimensions while others stagnate or decline, current evaluation practices—dominated by composite proficiency scores—would be structurally blind to the cost. Establishing whether such a dissociation exists, and whether it can be mitigated by pedagogical design, is a necessary precondition for responsible AI integration in oral language curricula.

Canale and Swain [4] model remains the clearest starting point for specifying what could be unevenly affected: communicative competence comprises grammatical, discourse, sociolinguistic, and strategic sub-competencies that are not interchangeable. Grammatical and discourse competence are amenable to automated assessment and correction with considerable precision. Sociolinguistic and strategic competence are different in kind: they depend on a real interlocutor with independent purposes, in an interaction that neither party fully controls. This distinction predicts where AI-mediated practice should be expected to help and where it should not.

This prediction connects to a broader theoretical literature on skill automaticity and deskilling that has received little attention in CALL research to date. Proceduralization accounts of skill acquisition hold that repeated practice under stable, predictable conditions produces fast, low-effort performance calibrated to the conditions of practice, not necessarily transferable to conditions requiring flexible, real-time adaptation. AI-mediated oral practice is, by design, a highly stable and predictable practice environment: the “interlocutor” does not interrupt unpredictably, does not require face-saving repair, and does not impose the interpersonal stakes of human interaction. Deskilling theory, from labour process research, offers a parallel warning: when a task is restructured so that a subset of its component skills is automated, proficiency in the non-automated remainder does not stay constant—it can atrophy from disuse, because the automated component now handles the cognitive load that previously forced practice of the whole skill. Applied to oral English instruction, this suggests that AI-mediated practice, left unstructured, may not simply fail to develop strategic and sociolinguistic competence—it may actively displace the practice opportunities that would otherwise develop it.

Recent empirical work is consistent with this account, though none has framed it in these terms. Studies confirm genuine gains from AI tools in pronunciation accuracy, fluency, lexical diversity, and output volume [3,5–10]—precisely the dimensions proceduralization theory predicts AI practice should improve. At the same time, multiple reviewers note a systematic asymmetry in what gets measured: studies of AI’s benefits overwhelmingly target dimensions amenable to automated assessment, while interactional and pragmatic dimensions receive comparatively little attention [1,11–13], an asymmetry that could obscure the dissociation this study investigates rather than demonstrate its absence. Kecskes and Dinh [14] reach a compatible conclusion from a pragmatics standpoint: ChatGPT demonstrates surface-level linguistic flexibility but does not engage in the genuine meaning negotiation characteristic of human-to-human pragmatic development.

No existing study, however, has tested this dissociation directly using a design capable of separating within-class developmental trajectories from between-group differences, nor has any proposed and tested a pedagogical response. This is the gap the present study addresses. Competence narrowing is defined here as the systematic process by which sustained AI-mediated oral practice, through concentration on automatable dimensions of communicative competence, progressively marginalises the conditions under which sociolinguistic competence, strategic competence, and pragmatic adaptability develop. Framed against proceduralization and deskilling theory, competence narrowing is not simply an empirical curiosity but a predictable consequence of restructuring a communicative task around a partial automation of its component skills—one that existing composite-score evaluation practices are not designed to detect.

The Spiral Synergy Framework (SSF) is proposed as a design response to this predicted mechanism. Rather than treating AI-mediated and interpersonal practice as substitutable, or leaving their relationship unstructured, the SSF sequences them as functionally distinct and complementary: AI-mediated practice is deliberately confined to consolidating the automatable sub-skills, freeing subsequent interpersonal tasks to demand precisely the unscripted, contingent performance that AI practice does not require. If deskilling theory is correct that skill atrophy follows from the removal of practice opportunities rather than from AI use per se, then reinstating those opportunities by design—rather than assuming they will occur incidentally—should be sufficient to interrupt the narrowing process, even without removing AI tools from the curriculum.

## Materials and methods

### Research design

This study employed a quasi-experimental, non-equivalent control group design with pre-test, mid-semester, and post-test measures across one academic semester (15 weeks). Two intact Business English classes were assigned to experimental (SSF) and control (conventional AI-assisted instruction) conditions. Three data strands were collected: (1) a 15-item Likert questionnaire at pre-test (T1); (2) monthly oral recordings scored on five communicative competence dimensions at T1, T2 (mid-semester), and T3 (end of semester); and (3) weekly structured classroom observation for the experimental class across all 15 weeks.

### Participants

Data collection took place over one academic semester, from 1 April 2026 (T1, pre-test) to 25 June 2026 (T3, post-test), with the mid-semester (T2) measurement on 20 May 2026. Participants were students enrolled in two parallel intact sections of a second-year Business English course at a higher vocational college in Jiangsu Province, eastern China. Class 241302 served as the experimental group (n = 36) and Class 241301 as the control group (n = 34), assigned by the institution prior to the study. All 34 control-class students completed both the T1 and T3 recordings; 4 of the 34 were absent for the T2 (mid-semester) recording, so T1 → T2 within-group comparisons for the control class are based on n = 30 (see Analytic plan; Missing data). All students were aged approximately 18–20 years and in their second year of a three-year higher vocational programme. Both classes were predominantly female, consistent with the gender distribution typical of Business English programmes at higher vocational colleges in China.

One additional T2 recording was obtained from a control-class student without a corresponding T1 baseline; this recording is included in the total count of T2 recordings collected (n = 31) but excluded from analyses requiring a baseline, including the linear mixed-effects models reported in Results (see Missing data). This accounts for the n = 30 (baseline-matched) versus n = 31 (total recordings collected) figures appearing at different points in this manuscript.

Prior English proficiency was assessed using CET-4 scores available for 25 experimental-class students (M = 459.1, SD = 47.9, range 373–573) and 26 control-class students (M = 456.8, SD = 53.0, range 364–560). An independent-samples t-test confirmed no significant between-group difference (t = 0.163, p = .871, d = 0.046). Pass rates (CET-4 ≥ 425) were 80% (experimental) and 73% (control). Both classes were taught by the same instructor (the author).

### Instruments

#### Questionnaire.

A 15-item bilingual Likert questionnaire (1 = strongly disagree to 5 = strongly agree) was administered at T1. Part A (AI Dependency, 8 items) showed marginal internal consistency (α = .66); Part B (Authentic Communicative Confidence, 7 items) showed acceptable reliability (α = .893). No post-test questionnaire was administered in this study cycle; this is acknowledged as a limitation and a priority for future research.

#### Oral recording tasks.

Monthly oral recording tasks were administered at T1 (Weeks 1–2), T2 (Weeks 8–9), and T3 (Weeks 16–17). All tasks shared the same format: individual performance, timed at two minutes, completed in class without scripts, notes, or AI tools. The same task type—impromptu situational expression—was used across all three time points, varying only in communicative scenario.

The T1 task asked students to give a nervous friend three specific pieces of advice for an upcoming job interview. The T2 task asked students to simulate a face-to-face meeting with a dissatisfied client as company representative—acknowledging the complaint, explaining the issue, and proposing a solution, eliciting a more demanding professional register under interactional pressure. The T3 task invited students to share, in a simulated class forum, the most important thing they had learned during the semester, based on genuine personal experience with no AI-generatable correct answer—maximally eliciting authentic improvisation and personal voice.

Recordings were rated by the researcher directly, using the five-dimension rubric shown in Table 1; NotebookLM (Google) was used only to transcribe and organise recordings for review, not to generate or suggest scores. Disfluency markers were not consistently preserved in transcription output, rendering ② repair counts non-comparable at T3; this dimension is excluded from T3 longitudinal analysis (see Limitations). T1 and T2 repair counts are based on unmodified transcripts and are reported for the within-group T1 → T2 comparison only.

**Table 1:** Five-dimension oral scoring rubric.

Dimension	Description	Scale
① Script dependency	Degree of scripted vs. spontaneous delivery	1 (fully improvised) – 5 (highly scripted)
② Repair	Frequency of verbal repair/reformulation	Count
③ Real-time responsiveness	Adaptive response to task demands	1 (minimal) – 5 (high)
④ Coherence	Logical organisation and connectedness	1 (minimal) – 5 (high)
⑤ Naturalness	Overall naturalness and fluency	1 (minimal) – 5 (high)

*① is reverse-coded prior to analysis (Improvisation = 6 − Script dependency) so that higher values indicate greater improvisation, consistent with the other four dimensions. Composite score = reverse-coded ① + ③ + ④ + ⑤ (range 0–20). ② retained as a separate process indicator.*

#### Classroom observation protocol.

A structured observation protocol was completed by the instructor within five minutes of each class session for the experimental class across all 15 weeks. The protocol recorded turn-taking frequency, peer eye contact, AI-script dependency rates, off-script anxiety, body language, and holistic communicative quality (1–5), accompanied by qualitative field notes on the most notable communicative behaviour, most engaging task segment, and instructional adjustments. Observation data function as within-group longitudinal description rather than between-group comparison.

### Intervention procedure

The SSF structured the experimental class’s oral instruction across three functional layers per instructional cycle. Layer 1 (AI Consolidation) deployed AI tools for foundational linguistic preparation—pronunciation, vocabulary, discourse organisation—explicitly oriented toward the interpersonal task to follow. Layer 2 (Bridging Tasks) activated sociolinguistic and strategic competence through role-plays, information-gap tasks, and discussions in which students required genuine interactional outcomes from their interlocutors. Layer 3 (Interpersonal Activation) engaged students in unscripted communicative tasks—professional simulations, cross-group debates, personal sharing—designed to elicit the full range of communicative capacities beyond what AI practice develops. Each cycle repeated at increasing complexity and decreasing AI scaffolding across the 15 weeks.

The control class received conventional instruction in which AI-assisted practice and interpersonal tasks were used without this structured sequencing.

### Analytic plan

Within-group changes from T1 to T2 were examined using paired-samples t-tests, reported descriptively (see Results). Between-group differences across the full T1–T3 period were examined using linear mixed-effects models (LMM) with random intercepts for student, with Group, Time, and their interaction as fixed effects; the Group × Time interaction was tested via likelihood-ratio comparison against a reduced model, separately for each of four rating-scale dimensions and the composite score. Benjamini–Hochberg correction was applied across these five outcomes. Simple T2-vs-T1 and T3-vs-T1 contrasts were extracted from the same model. As a sensitivity check, contrasts for the one outcome with non-normal model residuals were re-estimated using cluster-robust standard errors clustered by student. The original T1 → T3 ANCOVA and gain-score analysis (T1 composite as covariate, following confirmation that the homogeneity-of-regression-slopes assumption was met, interaction p = .165) is retained and reported alongside the LMM composite result for comparison across analytic approaches (see Results). Intra-rater test–retest reliability was assessed via ICC(2,1) on a stratified sample of recordings re-scored after a four-month interval (see Reliability analysis). Effect sizes are reported as Cohen’s d where applicable. Statistical software and version, and code used for the analysis, are reported in the Data availability statement below. α = .05; marginal effects (p < .10) are reported and labelled accordingly where they arise in the original gain-score and ANCOVA analyses retained for comparison.

### Use of AI tools

Claude (Anthropic, models Sonnet 4.5 and Sonnet 4.6) was used during manuscript preparation and revision in two capacities. First, as a writing assistant for drafting and structuring text, under the author’s direction and subject to the author’s final editorial judgment throughout. Second, during the major-revision process prompted by peer review, Claude assisted in constructing and executing the statistical reanalysis reported in this manuscript — including the linear mixed-effects models, Benjamini–Hochberg correction, cluster-robust sensitivity checks, missing-data analysis, and intra-rater reliability analysis (Results) — implemented in Python (see Data availability statement for software versions and code). All analytic choices (e.g., model specification, correction method, sample stratification for the reliability check) were made or approved by the author, who independently verified reported statistics against the underlying data (e.g., cross-checking computed baseline means against values in the original submitted manuscript) before incorporating them into the text. The author takes full responsibility for the accuracy, integrity, and originality of all content, including all statistical code and results.

### Ethics statement

This study was conducted as part of the author’s routine teaching practice in a Business English course at Jiangsu Maritime Institute. The oral recordings and questionnaire were initially collected for routine instructional assessment purposes and were subsequently analysed and reported for research purposes. The institution does not maintain a formal Institutional Review Board or human-research ethics committee for pedagogical research of this kind.

All course activities, including the oral recordings and questionnaire, were conducted through the institution’s standard learning management platform (Chaoxing/Xuexitong), on which instructors routinely collect and analyse student performance data to inform lesson planning and individualised, one-on-one instructional guidance as part of normal teaching practice at the institution; students were aware that their classroom data were collected and used by the instructor for this purpose. No separate written or verbal consent specific to this research study was sought, consistent with standard institutional practice governing platform-based instructional data at the time of data collection.

Participation in the oral recording tasks and questionnaire was not part of course grading or assessment, so students’ academic standing was not affected by their performance on, or participation in, these activities. All participating students were adults (approximately 18–20 years of age); no minors were involved.

Data were anonymised prior to analysis, and no individually identifying information is reported in the manuscript. Audio recordings are retained by the author and are not publicly available, in order to protect participant privacy (see Data Availability Statement); de-identified numerical scores derived from the recordings are reported in the manuscript and deposited in a public repository.

## Results

### Baseline equivalence

Questionnaire. Independent-samples t-tests confirmed no significant between-group differences on any of the 15 questionnaire items (all p > .05). Oral recordings showed the control class scored significantly higher on the composite (M = 12.59 vs. 11.53; t=−2.13, p = .037, d=−0.51) and on ③ responsiveness (p = .010, d=−0.63), ④ coherence (p = .006, d=−0.67), and ⑤ naturalness (p = .040, d=−0.50); the experimental class scored marginally higher on ① improvisation (p = .068, d=+0.44). This recording baseline inequivalence is addressed below both via the original ANCOVA and gain-score analysis and, as recommended during review, by modelling all three time points jointly so that the Group main effect absorbs the baseline offset directly (see Longitudinal analysis, below).

### Mid-semester recording (T2): within-group gains and early dissociation

Experimental class. Significant improvements on composite (Δ=+2.47, t = 5.43, p < .001), ③ responsiveness (Δ=+1.25, p < .001), ④ coherence (Δ=+0.94, p < .001), ⑤ naturalness (Δ=+0.72, p < .001), and ② repair (Δ=+0.92, p < .001). Critically, ① improvisation showed a significant decline (Δ=−0.44, t=−2.17, p = .037)—composite improvement concurrent with improvisation regression, constituting initial evidence of competence narrowing.

Control class (n = 30 with complete T1 and T2 data; see Participants). Significant composite improvement (Δ=+1.33, t = 3.81, p < .001), but ① improvisation showed no significant change (Δ=+0.13, p = .556). The improvisation decline observed in the experimental class was absent in the control class.

Between-group at T2. The baseline gap had closed: experimental composite (M = 14.00) narrowly exceeded control (M = 13.81, n = 31 available at T2), no significant difference (p = .684). The experimental class’s ③ responsiveness advantage approached significance (p = .096, d = 0.41).

### End-of-semester recording (T3): ANCOVA and gain-score analysis

Composite—ANCOVA. Controlling for T1 composite, the main effect of group on T3 composite was non-significant (p = .169). The gain-score t-test corroborated this (t = 0.592, p = .556). The SSF did not produce a significant overall composite score advantage by end of semester.

Dimension-level gain-score analysis. Table 2 presents gains by dimension and group.

**Table 2:** Gain scores (T3 − T1) by dimension and group with between-group comparisons.

Dimension	EXP-T1 M	EXP-T3 M	EXP gain	Ctrl-T1 M	Ctrl-T3 M	Ctrl gain	Diff	t	p	d
① Improvisation	2.28	1.53	−0.75***	1.85	1.44	−0.41**	−0.34	−1.329	.188	−0.318
③ Responsiveness	2.92	3.00	+0.08 ns	3.38	2.91	−0.47**	+0.55*	2.402	.019	0.574
④ Coherence	3.39	3.89	+0.50*	4.00	4.35	+0.35*	+0.15	0.526	.600	0.126
⑤ Naturalness	2.94	3.17	+0.22 ns	3.35	3.56	+0.21†	+0.02	0.074	.941	0.018
Composite /20	11.53	11.58	+0.06 ns	12.59	12.26	−0.32 ns	+0.38	0.592	.556	0.142

EXP n = 36, CTRL n = 34 (all students with complete T1 and T3 recordings; see Participants). ① is reverse-coded so that higher scores indicate greater improvisation (see Oral recording tasks); composite = reverse-coded ① + ③ + ④ + ⑤. ② excluded (transcript cleaning). Within-group significance (paired t vs. 0): † p < .10, * p < .05, ** p < .01, *** p < .001. Between-group gain: independent-samples t.

The single significant between-group difference was ③ real-time responsiveness (t = 2.402, p = .019, d = 0.574). The direction is theoretically consequential: the experimental class maintained responsiveness (Δ=+0.08, p = .646) while the control class showed a significant decline (Δ=−0.47, p = .002)—despite beginning the semester with higher responsiveness (M = 3.38 vs. 2.92). On ① improvisation, both groups declined significantly by end of semester (experimental Δ=−0.75, p < .001; control Δ=−0.41, p = .008) with no significant between-group difference (p = .188), indicating that improvisation narrowing occurred regardless of instructional condition. Composite scores showed no significant within-group change in either condition (experimental p = .916; control p = .369) and no significant between-group difference (p = .556). This gain-score comparison is retained here for continuity with the original analysis; a complementary analysis modelling all three time points jointly, with correction for the five outcomes tested, is reported in Longitudinal analysis below.

### Longitudinal analysis: Group × Time linear mixed-effects models

Following reviewer feedback, within- and between-group change across T1, T2, and T3 was additionally analyzed using linear mixed-effects models (LMM) with a random intercept for student. This approach models the full Group × Time structure directly, accommodates the baseline inequivalence noted above without a separate covariate-adjustment step, and uses all available observations for each student under maximum-likelihood estimation rather than requiring complete T1–T3 data (see Missing data, below) (Table 3).

**Table 3:** Omnibus Group × Time test, Benjamini–Hochberg-adjusted p, and simple contrasts (T2 vs. T1, T3 vs. T1), by outcome.

Outcome	χ²(2)	p	BH-adjusted p	T2 vs. T1 (β, p)	T3 vs. T1 (β, p)
① Improvisation	4.57	.102	.170	—	—
③ Responsiveness	10.35	.0057	.029	+0.80, p = .002	+0.55, p = .027
④ Coherence	2.81	.245	.245	—	—
⑤ Naturalness	4.62	.099	.170	—	—
Composite	3.58	.167	.209	—	—

For each of four rating-scale dimensions (① improvisation, ③ responsiveness, ④ coherence, ⑤ naturalness) and the composite score, a model with Group, Time, and their interaction as fixed effects was compared against a reduced model without the interaction, via likelihood-ratio test. Because five outcomes were tested, p-values were adjusted using the Benjamini–Hochberg procedure. ② Repair count was analyzed separately as a descriptive process indicator and is not included in this correction family (see below).

The only outcome whose Group × Time interaction survived correction was ③ responsiveness. The experimental class’s responsiveness rose relative to the control class both at T2 (β = 0.80, p = .002) and at T3 (β = 0.55, p = .027) relative to T1. Equivalently: the control class—despite beginning the semester with higher responsiveness (M = 3.38 vs. 2.92)—declined over the semester, while the experimental class held roughly steady. This is the same directional pattern reported above using the original T3 − T1 gain-score comparison (t = 2.402, p = .019, d = 0.574); the LMM reproduces it using all three time points jointly and shows it survives correction for testing five outcomes.

No other dimension’s Group × Time interaction reached significance after correction. The composite-score result (p = .167, BH-adjusted p = .209) is quantitatively close to the ANCOVA-based composite result reported above (p = .169), providing convergent evidence across two different analytic approaches that the SSF did not produce a detectable aggregate-score advantage.

② Repair count was zero-inflated (0 for the large majority of recordings; see Reliability analysis, below) and its omnibus interaction was not significant (χ²(2)=3.84, p = .146); given its distributional properties, this result is reported descriptively rather than treated as confirmatory.

### Sensitivity analysis

Because model residuals for the ③ responsiveness model departed from normality (Shapiro–Wilk p < .001, likely reflecting the discrete 1–5 rating scale), the T2-vs-T1 and T3-vs-T1 contrasts were re-estimated using cluster-robust standard errors (clustered by student), which do not rely on residual normality. Results were consistent with the LMM: T2 vs. T1, p = .003; T3 vs. T1, p = .016.

### Missing data

Independent-samples t-tests compared T1 baseline scores (all dimensions and composite) between the four control-class students missing T2 data and the 30 with complete T2 data; no significant differences were found on any dimension (all p > .62), providing no evidence that T2 non-completion was related to students’ baseline oral competence. We note the limited statistical power of this specific comparison given the small number of missing cases.

### Reliability analysis

All recordings were rated directly by the researcher using the rubric in Table 1; NotebookLM was used only to organize transcripts, not to generate or suggest scores (see Materials and methods). Because the study was not designed from the outset to include a second rater, reliability was assessed via intra-rater test–retest agreement: the same rater re-scored a stratified random sample of 45 recordings (22% of valid recordings, proportionally distributed across group and time point) after a four-month interval, with group membership withheld from the retest sheet. One recording could not be located for retest (final n = 44).

Intraclass correlation coefficients (ICC(2,1), absolute agreement) between original and retest scores were: ① improvisation, .77 [.60, .87]; ③ responsiveness, .72 [.50, .85]; ④ coherence, .91 [.84, .95]; ⑤ naturalness, .67 [.44, .81]. ② Repair count showed complete exact agreement (44/44) but minimal underlying variance. Independent-samples t-tests comparing the magnitude of original–retest discrepancies between groups, run separately per dimension, found no evidence of systematic group-related divergence (all p > .10)—i.e., no evidence that scoring drift was larger for one group than the other (see Limitations for the interpretive caveat regarding rater familiarity with participants).

### Classroom observation: longitudinal patterns

Classroom observation data (experimental class, Weeks 1–15) corroborate the recording-based findings in three patterns. First, communicative quality (1–5 holistic) showed a wave pattern: improvements in Weeks 3–8 were followed by dips in Weeks 9 and 12, consistent with the T2 peak / T3 partial-decline trajectory in the recording data.

Second, AI-script dependency did not decline monotonically. After falling from 100% (Weeks 1–3) to 25% (Week 8), dependency rose to 75% in Weeks 11 and 13–15. Off-script anxiety reached 100% in Weeks 9 and 15. These rebound episodes correspond temporally with the T2 → T3 decline in ③ responsiveness.

Third, qualitatively distinct phenomena emerged in Weeks 11–15: (a) Week 13—mechanical speech, students reading AI-generated content with flattened prosody; (b) Week 14—within-class polarisation, some students demonstrating confident scaffold-supported fluency while others could not initiate without a script; (c) Weeks 10 and 15—spontaneous student assertions of AI independence, followed by renewed dependency in subsequent sessions.

### Summary of key findings

Five findings, stated with the caution the design and measurement properties warrant: (1) Baseline inequivalence—the two intact classes differed significantly at baseline on the oral composite and three of five dimensions (control higher); this is addressed throughout by modelling all time points jointly rather than relying on a single covariate adjustment. (2) Responsiveness is the sole outcome showing a robust, corrected group difference—of five outcomes tested, only ③ responsiveness showed a Group × Time interaction surviving Benjamini–Hochberg correction (p = .029) and a cluster-robust sensitivity check; the experimental class maintained responsiveness while the control class declined, despite its baseline advantage. (3) No corrected evidence of a group difference elsewhere—① improvisation, ④ coherence, ⑤ naturalness, and the composite score showed no Group × Time interaction surviving correction; where narrowing is observed descriptively on these outcomes (e.g., improvisation declining in both classes), it does not differentiate the two instructional conditions. (4) Measurement reliability is moderate to good but reflects a single, familiar rater—intra-rater test–retest ICCs ranged from .67 to .91 across dimensions (.72 for responsiveness); because the rater had taught all participants for a semester, these estimates should be read as an upper bound on measurement consistency, not as equivalent to independent inter-rater reliability (see Limitations). (5) Classroom observation corroborates a cyclical, non-monotonic pattern of AI-dependency reduction and rebound rather than a stable, cumulative trajectory.

## Discussion

### Competence narrowing as an empirically observable pattern

The present study finds evidence consistent with competence narrowing as a describable pattern in this sample, though the design constrains how strongly that evidence can be interpreted. The experimental class’s T1 → T2 profile—a composite gain alongside an improvisation decline within the same students over the same interval—is descriptively consistent with the pattern the hypothesis anticipates: aggregate improvement co-occurring with a measurable cost at the dimension level, a cost that would be invisible to any assessment reporting only composite scores. This within-group pattern is reported descriptively rather than as a further hypothesis-tested effect; it was not part of the Group × Time interaction family subjected to multiplicity correction (see Results), and should be read as motivating rather than confirming the broader account below.

The full-semester (T1 → T3) data add an important qualification: improvisation declined descriptively in both conditions, and this dimension showed no Group × Time interaction surviving correction (BH-adjusted p = .170). Improvisation narrowing over the semester is therefore not attributable, on the present evidence, to the SSF condition specifically, nor can it be attributed to AI-assisted practice as such, since both classes used AI-assisted oral practice—the design compares two ways of organising that practice, not AI use against its absence (see Limitations). What the between-group analysis does support, at the level the corrected statistics allow, is a difference in a single dimension: ③ responsiveness.

### The SSF’s association with real-time responsiveness

Of the five outcomes examined, only ③ responsiveness showed a Group × Time interaction that survived correction for multiple comparisons (BH-adjusted p = .029) and remained significant under a cluster-robust sensitivity check that does not depend on the normality of model residuals. The experimental class maintained responsiveness across the semester while the control class declined, despite the control class’s higher baseline. This is consistent with—though it does not, on a non-randomised two-intact-class design, establish—a role for the SSF’s Interpersonal Activation layer, which specifically structures tasks requiring unscripted, contingent response. We present this as the study’s central empirical observation rather than as a demonstrated causal effect: treatment is confounded with class membership, there is no second experimental class to replicate the pattern, and the two conditions differ in how AI-assisted practice was organised rather than in whether AI was used at all. Any of these features, individually or jointly, could in principle contribute to the observed divergence.

The absence of a corrected composite-score effect (LMM p = .167, BH-adjusted p = .209; consistent with the original T1 → T3 ANCOVA, p = .169) should be read alongside the responsiveness finding rather than as a separate, conflicting result. Composite scores aggregate dimensions with different trajectories—in this study, a coherence gain common to both conditions and an improvisation decline common to both conditions move in offsetting directions, leaving the aggregate comparatively flat in both groups. A between-group difference confined to one dimension is therefore compatible with a null composite result; the two findings are not in tension. This pattern illustrates why the study’s own analytic plan gave dimension-level comparison equal or greater weight than the composite score from the outset (see Analytic plan), and it carries a broader implication for how AI-integrated instruction is evaluated: composite-score comparisons alone may not detect dimension-specific reorganisation of this kind.

### Cyclic rebound and its implications for task design

The recurring rebound pattern—reduced AI dependency followed by anxiety spikes and renewed script reliance—is not visible in three-point recording data alone. The 15-week classroom observation record (experimental class only; see Limitations) identifies at least four distinct rebound episodes. Descriptively, this suggests that whatever supports reduced AI dependency needs to be sustained across the full semester rather than front-loaded. The Week 14 polarisation further suggests uneven effects within the class, with some students more vulnerable than others to dependency reversion—consistent with graduated rather than abrupt scaffold removal, though this observation comes from a single class with no comparison condition and should be treated as a hypothesis for future work rather than a finding.

Theoretically, the cyclic pattern is compatible with AI dependency functioning less like a habit gradually extinguished and more like a default response mode that re-activates under interactional pressure—a plausible account for a design of this kind, though not one this study can adjudicate against alternative explanations.

### Limitations

Several limitations qualify how the findings above should be read.

**Design confound.** The two conditions were two intact classes rather than randomly assigned individuals or replicated class-level units: treatment is completely confounded with class membership, and there is no class-level replication of either condition. Both classes received AI-assisted oral instruction; the comparison is between two ways of organising that instruction (SSF sequencing versus conventional integration), not between AI use and its absence. The between-group responsiveness difference reported above should accordingly be read as an association observed under this design, not as a demonstrated causal effect of the SSF. Baseline inequivalence on the oral composite and three of five dimensions (control higher; see Baseline equivalence) further means that, notwithstanding the longitudinal modelling approach adopted here, unmeasured differences between the two classes cannot be ruled out as contributing to the pattern observed.

**Measurement reliability.** All recordings were rated directly by the researcher, using NotebookLM only to organise and review transcripts, not to generate or suggest scores. Because the study was not designed from the outset to include a second rater, reliability was assessed via intra-rater test–retest agreement rather than independent inter-rater agreement (see Reliability analysis): the same rater re-scored a stratified sample of recordings after a four-month interval, blind to group membership. Resulting ICCs were moderate to good (.67–.91 across dimensions; .72 for responsiveness), but because the rater had taught all participants for a semester, apparent stability across the interval may partly reflect recognition of familiar students’ prior performance rather than fully independent re-judgment. These estimates should therefore be read as an upper bound on measurement consistency, not as equivalent to independent inter-rater reliability. An attempt to incorporate an independent second rater from the same teaching team was undertaken during revision but was not retained: it surfaced scoring-direction errors and range-restricted scoring in the resulting data, and sharing recordings beyond the instructor also exceeded the scope of the data-use notice originally given to participants (see Ethics statement). Relatedly, ② repair count proved poorly suited to standard reliability statistics given its heavily zero-inflated distribution, and is reported descriptively rather than as a confirmatory outcome throughout.

**Single-observer classroom data.** Classroom observation was conducted for the experimental class only, by the same researcher who also taught and rated recordings for both classes, raising the potential for observer-expectancy effects that this design cannot rule out.

**Transcript quality.** T3 transcript cleaning rendered ② repair counts non-comparable to earlier time points for that specific analysis.

**Generalisability.** The study was conducted at a single higher vocational college with two classes (n = 36, n = 34); as is common in classroom-based quasi-experimental research, individual-level randomisation was not feasible in this institutional setting, and generalisation to other populations requires further research. No post-test questionnaire was administered; this is identified as a priority for future work.

**Multiple comparisons.** Five outcomes (four dimensions and the composite) were tested for a Group × Time interaction and corrected using the Benjamini–Hochberg procedure; only responsiveness survived. Earlier within-group and simple-contrast comparisons reported in the Discussion above were not subjected to this same correction and should be read as descriptive rather than confirmatory.

## Conclusions

Three conclusions follow from this study, stated with the caution the design and measurement properties warrant. First, a dissociation between aggregate and dimension-level oral competence measures is observable in this sample: within the experimental class, a composite gain co-occurred with an improvisation decline at mid-semester, and by end of semester improvisation had declined in both classes while the composite score had not moved significantly in either. Aggregate oral scores did not reveal this dimension-level activity.

Second, of the outcomes examined, real-time responsiveness is the one dimension on which the two classes diverged in a way that survived correction for multiple comparisons: the experimental class maintained responsiveness while the control class—despite a higher baseline—declined. Given the non-randomised, two-intact-class design, this is reported as an association warranting further investigation under better-controlled conditions, not as a demonstrated causal effect of the SSF, and not as evidence that AI-assisted instruction as such produces competence narrowing, since both classes used AI-assisted practice.

Third, the 15-week observation record (experimental class) documents a recurring rather than cumulative pattern of AI-dependency reduction, with multiple rebound episodes across the semester; this suggests that whatever supports sustained autonomous performance may need to be maintained throughout a course of instruction rather than delivered as a front-loaded intervention, though this observation is descriptive and drawn from a single, non-comparison class.

For practitioners, these findings suggest that evaluation of AI-integrated oral instruction should consider dimension-level as well as aggregate measures, since composite stability is compatible with dimension-level change that aggregate metrics alone would not reveal. For researchers, competence narrowing—operationalised through multidimensional assessment and longitudinal classroom observation—offers a tractable empirical framework for further investigating what AI-mediated practice develops and what it leaves behind, provided future designs incorporate randomisation or class-level replication and independent, blinded rating from the outset.
